# Effects of Whole Body Vibration and Resistance Training on Bone Mineral Density and Anthropometry in Obese Postmenopausal Women

**DOI:** 10.1155/2014/702589

**Published:** 2014-06-18

**Authors:** Moushira Erfan Zaki

**Affiliations:** Medical Research Division, Biological Anthropology Department, National Research Centre, El-Buhouth Street, Dokki, Giza, Egypt

## Abstract

*Objective*. The aim of this study was to evaluate the impact of two exercise programs, whole body vibration and resistance training on bone mineral density (BMD) and anthropometry in obese postmenopausal women. *Material and Methods*. Eighty Egyptian obese postmenopausal women were enrolled in this study; their age ranged from 50 to 68 years. Their body mass index ranged (30–36 kg/m^2^). The exercise prescription consisted of whole body vibration (WBV) and resistance training. Bone mineral density (BMD) and anthropometrical parameters were measured at the beginning and at the end of the study. Changes from baseline to eight months in BMD and anthropometric parameters were investigated. *Results*. BMD at the greater trochanter, at ward's triangle, and at lumbar spine were significantly higher after physical training, using both WBV and resistive training. Moreover, both exercise programs were effective in BMI and waist to the hip ratio. Simple and multiple regression analyses showed significant associations between physical activity duration and BMD at all sites. The highest values of *R* ^2^ were found for the models incorporating WBV plus BMI. *Conclusion*. The study suggests that both types of exercise modalities had a similar positive effect on BMD at all sites in obese postmenopausal women. Significant association was noted between physical activity and anthropometric variables and BMD measures at all sites.

## 1. Introduction

One aspect of health that is particularly important for postmenopausal women is bone mineral density. Decreasing estrogen concentrations after menopause can cause a decline in bone mineral density, which can lead to osteoporosis. Physical exercise is considered as an effective strategy for the prevention and management of postmenopausal complaints. Aerobics, weight bearing, and resistance exercises were all effective in increasing BMD. However, bone stress induced by vigorous weight-bearing activities can increase the risk of injuries, particularly in the elderly. Therefore, alternative strategies with a lower risk of injury are sought and usually included in the medical advice [1].

Resistance exercise is designed to improve muscle strength, power, and endurance. However, many studies reported that it places also heavy loads on the skeleton during a training session, which increases BMD [2]. Some studies also reported its positive effect on body composition [3]. The exercise dose in resistance exercise training is usually described by the magnitude of resistance, the number of repetitions the resistance is moved in a single set of exercise, the number of sets done, and the length of the resistance training programs.

Vibration is most often considered as an etiologic factor in low back pain as well as several other musculoskeletal and neurovestibular complications, but recent experiments indicate that extremely low-level mechanical signals delivered to the bone in sufficient frequency range can be strongly anabolic. If these mechanical signals can be effective and noninvasively transmitted into the standing human to reach those sites of the skeleton at the greatest risk of osteoporosis, such as the hip and lumbar spine, then vibration could be used as a unique, nonpharmacological intervention to prevent or reverse bone loss [4]. Whole body vibration (WBV) is a new type of exercise that has been increasingly tested for the ability to prevent muscular atrophy, bone fractures, and osteoporosis. Compared to traditional training regimes, WBV needs significantly less time and, therefore, could be expected to reach a higher compliance in previously inactive patients [5–7].

In WBV training, the subject stands on a platform that generates vibrations with certain amplitude and frequency. These mechanical stimuli are transmitted to the body, where they load the bone and also stimulate sensory receptors (most likely muscle spindles) and so enlarge the drive to alpha motor neurons (motor units) via the monosynaptic stretch reflex, and hence initiate muscle contractions. The combination of a sedentary lifestyle and impaired functional status could lead to further reduction in physical activity level, thereby triggering a vicious cycle of physical deconditioning, bone loss, and falls. Although the importance of physical activity is clearly emphasized by most guidelines, some of these fail to address what its desirable exercise and duration are. The role of exercise in obese postmenopausal women and associated benefits on bone health has been less explored. The aim of this study was to investigate the effect of whole body vibration and resistance training on bone mineral density and anthropometric parameters in obese postmenopausal women. Their body mass index ranged 30–36 Kg/m^2^.

## 2. Subjects and Methods

Eighty physically untrained postmenopausal women, their ages ranged from 50 to 68 years and their body mass index ranged 30–36 Kg/m^2^ were randomly selected and divided into two groups. The exercise group underwent resistive training, three times a week for eight months. The WBV group trained for 20 minutes three times a week for eight months.

Both groups were evaluated before and after the study period. The inclusion criteria were female, greater than two years menopausal, not on estrogen replacement therapy, stable weight maintenance, estimated daily calcium intake of 500 mg/day or more, resting blood pressure ≤160/100 mm Hg, ability to follow the protocol, and free from disease or medication known to affect bone metabolism.

Exclusion criteria included acute hernia; thromboembolism, current smoking status, any use of steroids; history of severe musculoskeletal problems; diabetes mellitus; subjects engaged in high-impact activity at least twice a week (any weight-bearing activity or exercise more intense than brisk walking), cardiovascular disease, endogenous osteosynthetically material, knee or hip prosthesis, epilepsy, pacemaker, history of low energy or nontraumatic fractures, malignancy, and renal, liver, or thyroid disorders. The participants were randomly divided into two equal groups. Whole body vibration group consisted of forty women who participated in a supervised training program using whole body vibration, three times a week for eight months. Whole body vibration apparatus (model OMA-701A, made in China) was used for the whole body vibration program by reciprocating vertical displacements on the left and right side of a fulcrum. The WBV group received vibration training three times per week. The session started with an initial 5–10 minutes warm-up phase consisting of stretching exercises of the quadriceps, hamstring, and calf muscles. During the first session of training, the WBV group performed three sets of 1 minute vibration with a frequency of 16 Hz of vibration stimulus, separated by 1-minute resting periods. The training load increased systematically during the following sessions, increasing by one set every session until the 10 sets of WBV that is considered to be the load of this intervention. The resting period between sets was 1 minute. Resistive training program used free weights in the form of sand bags for large muscle groups of the lower limbs and different graduations for the trunk muscles. All subjects started the exercise program at 10-repetition maximum load for a given exercise for a total of 10 repetitions (one set). After one entire circuit (consisting of 8 sets, one set for each muscle group) was completed, a second circuit of exercises was performed on lower limb exercises. To maintain the appropriate intensity for these exercises, muscle testing was repeated for these muscles every 2 weeks. So, adjustments in weights (for the lower limb muscles) or graduations (for the trunk muscles) were made every 2 weeks throughout the duration of the study to continue increases in strength. The weights were added to or replaced with heavier weights or graduations when the person could achieve >10 repetitions. Subjects alternated between upper and lower body exercises to minimize fatigue, with approximately 2-minute rest between exercises with no rest between repetitions. The session duration was one hour. The resistance training group consisted of forty women who underwent the resistance training program, three times a week for eight months, with at least one day of rest between two sessions. Sand bags of different weights (1/2, 1, and 2 kilos) made in Germany were used. Dual Energy X-ray Absorptiometry (DXA) technique was used to measure BMD of the left femoral neck, greater trochanter, ward's triangle, and anterior-posterior (AP) lumbar spine (L2-L4). Body weight, height, and waist and hip circumferences were measured. Body mass index (BMI) and waist to the hip ratio (WHR) were calculated.

Distributions of continuous variables were examined for skewness and kurtosis. All results are presented as mean ± SD. Student's* t*-test was used for the analysis of data with a Gausian distribution. The data with non-Gausian distribution were compared with Mann-Whitney* U* test. The comparison was made by paired* t*-test to determine the probability levels for a difference in mean value between the results observed before and after the period of eight months in each group. The unpaired* t*-test was used to compare the significance of difference between the two groups (whole body vibration group and resistance training group). The chi-square test was used to compare the differences of categorical variables. ANOVA was used to evaluate mean of percent changes in BMD at all sites. The significant *P* value was <0.05. Simple linear regression was used to assess the relationship between physical activity variables and BMD at all sites.

The variables showing significant correlation with simple linear regression were then included in a multiple regression analysis with physical activity, duration of practice, age, body weight, BMI, and WHR as independent variables, to find out which of them were best correlated with BMD. All analyses were performed with SPSS version 17. Ethical approval and appropriate informed consent were obtained from all subjects.

## 3. Results

The studied sample contained eighty obese postmenopausal women. They were divided into two equal groups, the whole body vibration (WBV) group and resistance exercise group. The mean age of the WBV group was 57.34 ± 5.3 years old, and 56.95 ± 4.1 years old for the resistance exercise group. All participants continued the exercise program for 8 weeks. The mean value of menopausal years was 13.05 ± 6.31 for the WBV group and 9 ± 6.25 for the resistance group. The mean value of body mass index (BMI) was 35.54 + 6.51 for the WBV group while for resistive group it was 34.63 + 5.69. There was no statistical significant difference between the two groups in age, BMI, menopausal years, and BMD of measured sites at baseline (Table 1).  Table 2 shows mean values of BMD at femoral neck, greater trochanter, ward's triangle, and anterior-posterior (AP) lumbar spine (L2-L4), and percent changes in BMD. There was a statistical significant increase of BMD at the greater trochanter, ward's triangle, and lumbar spine for WBV group and resistive group. Furthermore, no statistical significant difference was observed in percent changes of BMD at all sites for both types of exercises.  Table 3 shows that there was a statistical significant decrease of adjusted BMI for diet and physical activity, whereas WHR for both groups. In simple regression, it was found that practice duration of both physical activity types had a strong correlation with BMD at all sites (Table 4). The main results of multiple regression analysis are featured in Table 5. The coefficient of determination (*R* ^2^) depicts the fraction of total variance of the BMD dependent variable, which is explained by the models. The highest values of *R* ^2^ were found for the models incorporating WBV plus BMI.

## 4. Discussion

The most common cause of osteoporosis is the decrease in the female sex hormone, estrogen, which occurs following menopause. An increase in bone resorption, which is associated with a rise in the number of osteoclasts, is correlated with the loss of estrogen. This increase in osteoclasts is caused by an increase in the cytokines that regulate the production of osteoclasts. It is believed that estrogen, either directly or indirectly, regulates the production of these cytokines.

Furthermore, under normal circumstances, the peak bone mass of women is lower than that of men, leading to a higher incidence of osteoporosis in postmenopausal women [8, 9]. Exercise is recommended as a preventative measure for osteoporosis. Some studies indicated that low-level mechanical signals induced via whole body vibration is anabolic to bone and thus may be used, noninvasively, as a form of “passive” exercise to positively influence skeletal status [10]. Weight loss typically reduces bone mineral density. One mechanism through which physical activity could increase bone strength is by increasing muscle mass. Lean body mass is thought to increase bone mineral density through mechanical loading of the skeleton. Muscle, a component of lean mass, is important because muscle contractions exert a greater force on bones than do other weight-associated gravitational forces. In postmenopausal women, adipose tissue is the main site of androgen conversion to estrogen by the enzyme aromatase. As overweight and obese postmenopausal women lose body fat, their serum estrogen concentrations decrease. Furthermore, body weight, particularly fat mass, contributes to the skeletal load and is therefore an important factor in increasing bone density and reducing bone turnover.

Exercise may preserve or increase BMD even while reducing fatness [11]. Although exercise remains the most readily available and generally accepted means of curbing weight gain, compliance is poor. The results of the present study revealed that BMD of greater trochanter, ward's triangle, and lumbar spine was significantly increased in WBV training group. Some studies found no effect of the vibration intervention on the bone turnover rates, indicating that its positive impact on BMD did not result from reduced bone resorption [12]. While others reported that whole body vibration caused a decrease in osteoclastic resorption on the trabeculae, suggesting that one or more signals act to inhibit the catabolic response [13]. There are a number of possible explanations for the experimental observation that whole body vibration is anabolic to bone, especially in the cancellous bone of postmenopausal women. Several fluid components intermixed within the intratrabecular space are present in bone. Bone marrow is the chief fluid constituent, but blood, lymph cells, and interstitial fluid are also present in varying amounts. Dynamic loading creates fluid movement in the bone's structural network, which in turn generates shear stresses on the plasma membranes of resident osteocytes, bone lining cells, and osteoblasts. Bone cells are highly sensitive to fluid shear stresses [4, 14, 15].

Similarly, the results of the present study revealed that BMD of greater trochanter, ward's triangle, and the lumbar spine was significantly increased in resistive exercise training group. Our results are in agreement with previous studies reporting that resistance training had the significant positive effect on the lumbar spine and total hip BMD [16, 17]. However the long treatment period enhanced results more than the current study. The current study results supported by the work of other studies concluded that the findings for high-intensity resistance training effects on the lumbar spine were significant [18]. A nonsignificant positive effect was also evident in the total hip. In contrast, results in femoral neck were inconsistent. Positive effects of resistance training in this study confirm the findings of Nickols-Richardson et al. [19] who trained young women using isokinetic resistance training for 5 months. However, they concluded that resistance training imparted benefit of total body bone mineral content also beside the site-specific bone mineral content. Resistive exercise lowers intramuscular lipids in skeletal muscle presumably by activating lipolysis. Like lipolysis in subcutaneous adipose tissue, catecholamines can activate lipolysis in the intramuscular lipid stores [20]. Although it has been reported that intramuscular lipids are utilized during resistive exercise, it is presumed that the immediate and glycolytic energy systems provide most of the energy during resistive exercise, which leads to a significant lowering of glycogen stores in recruited muscle. It has been suggested that the increase in fat oxidation after a resistive exercise bout allows for available glucose to be utilized for glycogen restoration as the skeletal muscle switches to utilizing the elevated fatty acids as the primary energy source. Whole body fat oxidation, as indicated by a significant reduction in the respiratory exchange ratio, was indeed increased following resistive exercise [21, 22]. Therefore, resistive training may help to attenuate weight gain and improve body composition and this may, in part, occur through the mechanisms of increasing energy expenditure, subcutaneous lipolysis, and whole body fat oxidation. The present study results coincided with those of Ryan et al. [16] who found that 16 weeks of strength exercises caused a small but significant decrease in body weight and body mass index in postmenopausal women. The findings of this study contrasted with those of Elliott et al. [23] who found after eight weeks of resistance training nonsignificant decrease in the body mass, percentage body fat, waist to hip ratio, and body mass index. The shorter treatment period may be the cause of this discrepancy.

The main results of multiple regression analysis showed that the highest values of *R* ^2^ were found for the models incorporating WBV plus BMI. Moreover, the present data showed that body mass index (BMI) and waist to the hip ratio (WHR) were reduced significantly after exercise in both groups. This is in agreement with a study suggesting that BMI is inferior to body weight as a predictor of BMD [24]. For simple linear regression, duration of physical activity of WBV and resistive training are strong predictors for BMD at all sites. While it has been suggested that high-intensity resistance training has site-specific effects on BMD; namely, it increases lumbar spine, but not femoral neck BMD [25].

Meta-analysis by James and Carroll reported changes in FN and LS BMD for high-impact only protocols as well as combined impact/resistance training protocols in premenopausal women [26]. Simple meta-regression analyses resulted in several noteworthy associations that may be appropriate for future investigation. Specifically, there was a trend for greater increases in FN BMD with shorter exercise interventions as well as a statistically significant association between increases in FN BMD and fewer days per week of exercise. On possible explanation for the negative associations observed may have to do with the loss of calcium from excessive exercise [27].

Life style modification at the transition of menopause will go long way in preventing weight gain during this metabolically vulnerable period which will help in primary and secondary prevention of several chronic diseases and premature death beside keeping women physically and mentally fit in her menopause. Optimal physical activities are necessary for increasing bone mass and thus perhaps reducing the risk of osteoporosis. So intervention education program for the importance of dietary and healthy lifestyle practices including physical activity, adequate calcium intake, and no cigarette smoking must be considered. An understanding of how knowledge, attitudes, and practices of modifiable factors towards bone health status is a particularly important strategy for formulating appropriate, effective, and innovative health and nutritional intervention programs to maximize higher bone mass accretion. More nutritional promotion and education are required to stress the necessity of proactive healthy lifestyle modifications during early growing lifespan such as during childhood, adolescence, and young adulthood in order to prevent the rapid bone mass and consequently the risk of osteoporotic fractures later in life.

Short-term weight loss intervention studies, which are typically 3–6 months long, have demonstrated significant reductions in total body or regional BMD; others have reported increased BMD following weight reduction [28].

The main finding of this study was that both the vibratory exercise on a reciprocating plate and resistive training had similar effects on BMD at all sites. In addition, both types of exercise were effective in improving BMI and WHR. In conclusion, both HBV exercise and resistance training are associated with higher BMD and lower BMI and WHR in obese postmenopausal women. Moreover, physical activity duration showed significant positive association with BMD values at all sites.

## Figures and Tables

**Table 1:** Baseline characteristics of participants for the WBV and resistive exercise groups.

Variable	WBV group	Resistive group	*P* value
	Mean ± SD	Mean ± SD	
Age (year)	57.34 ± 5.3	56.95 ± 4.1	0.45
BMI (kg/m^2^)	35.54 ± 6.51	34.63 ± 5.69	0.972
Menopause (year)	13.05 ± 6.31	9 ± 6.25	0.072
BMD of Fem neck	0.7878 ± 0.109	0.8030 ± 0.109	0.661
BMD of Troch	0.649 ± 0.112	0.659 ± 0.095	0.776
BMD of Wards tri	0.585 ± 0.118	0.648 ± 0.142	0.139
BMD L2-L4	0.984 ± 0.146	0.913 ± 0.113	0.092

BMD: bone mineral density; Fem neck: femoral neck; Troch: greater trochanter; Wards tri = wards triangle, L: lumbar spine; SD: standard deviation; *P* value: level of significance.

**Table 2:** Mean and standard deviation of bone mineral density for the WBV and resistive exercise groups before and after the study.

BMD	Before Mean ± SD	After Mean ± SD	*t*-value	*P* value	% change Mean ± SD
Fem neck			0.75		
WBV	0.788 ± 0.109	0.789 ± 0.11		0.65	**0.98 **± **0.68**
Resistive	0.813 ± 0.109	0.822 ± 0.12	0.35	0.13	**0.88** ± **0.59**
Troch			1.85		
WBV	0.639 ± 0.112	0.699 ± 0.11		0.05	**1.03** ± **0.84**
Resistive	0.659 ± 0.095	0.669 ± 0.083	1.47	0.04	**1.05** ± **0.99**
Wards tri			1.48		
WBV	0.581 ± 0.118	0.675 ± 0.128		0.03	**1.16** ± **0.91**
Resistive	0.631 ± 0.142	0.687 ± 0.147	1.98	0.04	**1.08** ± **0.93**
BMD L2-L4			1.87		
WBV	0.954 ± 0.146	0.997 ± 0.142		0.04	**1.04** ± **0.89**
Resistive	0.912 ± 0.113	0.939 ± 0.115	2.44	0.02	**1.02** ± **0.87**

BMD: bone mineral density; Fem neck: femoral neck; troch: greater trochanter; Wards tri = ward's triangle, L: lumbar spine, SD: standard deviation; *P* value: level of significance.

**Table 3:** Mean and standard deviation of anthropometric measurements for the WBV and resistive exercise groups before and after the study.

	Group	Before Mean ± SD	After Mean ± SD	*t*-value	*P* value
BMI	WBV	35.54 ± 6.51	31.72 ± 4.62	1.85	0.04
	Resistive	34.63 ± 5.69	32.19 ± 4.29	1.91	0.02

WHR	WBV	0.88 ± 0.08	0.87 ± 0.06	0.85	0.14
	Resistive	0.89 ± 0.06	0.81 ± 0.05	1.97	0.01

WHR: waist to hip ratio; SD: standard deviation; MD: mean difference; *P* value: level of significance.

**Table 4:** Simple linear regression results for WBV and resistive exercise types in obese postmenopausal women.

Independent variables	BMD of femoral Neck	BMD of greater trochanter	BMD of wards triangle	BMD of L2-L4
Duration of WBV (minutes per week)	*R* ^2^ = 0.568	*R* ^2^ = 0.668	*R* ^2^ = 0.609	*R* ^2^ = 0.968
	*P* < 0.04	*P* < 0.04	*P* < 0.05	*P* < 0.05

Duration of resistive (minutes per week)	*R* ^2^ = 0.598	*R* ^2^ = 0.578	*R* ^2^ = 0.577	*R* ^2^ = 0.545
	*P* < 0.04	*P* < 0.04	*P* < 0.02	*P* < 0.03

*R* ^2^: Pearson's coefficient of correlation. Values of *P* indicate the probability of the slope between the independent and the dependent variables being nonsignificantly different from zero.

**Table 5:** Multiple regression results for anthropometry and physical activity in obese postmenopausal women.

Independent variables	BMD of femoral Neck	BMD of greater trochanter	BMD of wards triangle	BMD of L2-L4
WBV and BMI	*R* ^2^ = 0.599	*R* ^2^ = 0.668	*R* ^2^ = 0.819	*R* ^2^ = 0.765
	*P* < 0.002	*P* < 0.004	*P* < 0.005	*P* < 0.002

Resistive and BMI	*R* ^2^ = 0.498	*R* ^2^ = 0.588	*R* ^2^ = 0.533	*R* ^2^ = 0.544
	*P* < 0.04	*P* < 0.04	*P* < 0.02	*P* < 0.03

*R* ^2^: coefficient of determination. Values of *P* correspond to the probability of *R* ^2^.
